# A comparative analysis of postoperative visual outcomes following cataract surgery with different brands of Monofocal Intraocular Lenses

**DOI:** 10.12669/pjms.40.1.8157

**Published:** 2024

**Authors:** Muhammad Saleh Memon, Tauseef Mahmood, Umair Qidwai, Shahid Ahsan, Muhammad Arslan, Muhammad Faisal Fahim

**Affiliations:** 1 Muhammad Saleh Memon, FRCS(Eden) Department of Ophthalmology, Al Ibrahim Eye Hospital, Isra Postgraduate, Institute of Ophthalmology, Karachi, Pakistan; 2 Mr. Tauseef Mahmood, M.Sc. (Statistics) Department of Research, Al Ibrahim Eye Hospital, Isra Postgraduate, Institute of Ophthalmology, Karachi, Pakistan; 3 Dr. Umair Qidwai, FRCOphth, FRCS, FCPS, MBBS Department of Ophthalmology, Al Ibrahim Eye Hospital, Isra Postgraduate, Institute of Ophthalmology, Karachi, Pakistan; 4 Dr. Shahid Ahsan, M. Phil (Bio), M. Phil (NCD), PhD fellow Department of Biochemistry, Jinnah Medical & Dental College, Karachi, Pakistan; 5 Mr. Muhammad Arslan, (MCSW) Department of Research & Excellence, Al-Tibri Medical College, Karachi, Pakistan; 6 Mr. Muhammad Faisal Fahim, M.Sc. (Statistics) Department of Research, Bahria University of Medical and Dental College, Karachi, Pakistan

**Keywords:** Phacoemulsification, Intraocular lens, Visual acuity

## Abstract

**Objective:**

To compare visual outcomes after cataract surgery using three intraocular lenses (IOL) of different prices but similar properties.

**Methods:**

A comparative study with retrospective data of patients operated for phacoemulsification with monofocal IOL implants was carried out at Al-Ibrahim Eye Hospital (AIEH) from April 2021 to Feb 2022. Patients with diabetes, any preoperative ocular morbidity and unclear diagnosis were excluded from the study. Pre and post-operative best corrected visual acuity (BCVA) on 1^st^ day, 7^th^ day and 4-6 weeks were analyzed. IOLs were categorized on the basis of price into economical, standard and premium lenses. To minimize surgical bias, data was further stratified on the basis of surgical expertise.

**Results:**

Data of 3237 patients was analyzed. Economical lens (A) was implanted in 2647, standard (B) in 254 and premium (C) in 336 patients. On average BCVA (6/6 to 6/12) was achieved in 88.2% of patients. No significant difference was found at third follow up among BCVA of three IOls operated by senior surgeon, χ^2^_(2)_ = 3.216, p = 0.20, with median (IQR) is 0.2(0.2) logMAR for Group-A, 0.1(0.2) logMAR for Group-B and 0.2(0.1) logMAR for Group-C. When results of the rest of the surgeons was considered, significant difference was found among BCVA at 3^rd^ follow-up, χ^2^_(2)_ = 6.661, p = 0.036, with median (IQR) is 0.3(0.2) logMAR for Group-A, 0.2(0.1) logMAR for Group-B and 0.2(0.3) logMAR for Group-C.

**Conclusion:**

When surgical factors mainly, surgeon bias is removed, all three types of monofocal IOL had similar visual outcomes.

## INTRODUCTION

Cataract is one of the major causes of avoidable blindness globally^1^ and cataract surgery includes the removal of clouded natural lens and replacing it with an intraocular lens (IOL).^1^ It is one of the most frequently conducted ophthalmic procedures worldwide.^2^

The last few decades have witnessed notable developments in cataract surgery and IOL technology,^3^ leading to the rise of numerous brands and designs. Among wide variety of IOL used for cataract surgery, monofocal IOLs are broadly used due to their predictable outcomes^4^ and reliability in correcting vision at a single focal point. Each IOL brand offers unique optical properties,^5^ materials, and designs, which have sparked the interest of ophthalmic surgeons as well as ophthalmic researchers to examine how these variations impact postoperative visual outcomes.

The postoperative visual outcomes is one of the fundamental parameter to access the success of cataract surgery.^6^ There have been numerous comparative studies.^7^ Multifocal versus Monofocal, tinted vs. non-tinted, aspheric vs. spherical, multi- piece vs. single piece, biomaterial A (e.g. acrylic) vs. biomaterial B (e.g. silicone), sharp vs. round edged and hydrophobic versus hydrophilic.^8^,^9^ The literature is silent about the comparative studies based on the quality of the vision related to the price of the lenses.

The cost of cataract surgery varies highly in Pakistan, depending upon the hospital,^10^ IOL used and the rate of exchange. Nearly 70% of the population in Pakistan have to pay out of pocket to access the health services.^11^,^12^ In our hospital (AIEH, non-profit organization) cost of cataract surgery with monofocal IOLs ranges from PKR 12000 ($40) using unbranded lens to PKR 66000 ($220) using branded IOLs. With this marked difference in the price, it is difficult for service providers to councel patients about which lens to be used for surgery. On the other hand, patient needs assurance of good postoperative results with the economical lens.

No study so far has been conducted to compare the post-operative visual outcomes of the patients implanted with economically priced and costly lenses with similar properties. The study aimed at filling this gap by comparing the visual outcomes of three different monocular IOLs used at Al-Ibrahim Eye Hospital (AIEH) during the periods April 2021 to Feb 2022.

## METHODS

A comparative study with retrospective data was carried out at Al-Ibrahim Eye Hospital (AIEH) from April 2021 to February 2022.

### Ethical approval

It was taken from Hospital’s Research Ethical Committee and study protocol number was REC/IPIO/2021/075.

### Inclusion & Exclusion Criteria

Records of n=3237 patients were retrieved from Hospital Information Management System (HIMS) who met the inclusion criteria. Patients operated with phacoemulsification were considered for analysis. Availability of complete records, cases without any intraoperative complication and preoperative ocular morbidity were included in the study. Diabetic patients, missing or incomplete records, unclear diagnosis and patients who failed to attend at any visit were excluded from the study.

Three categories of lenses used were commercially available unbranded IOL, Tecresoft model FLEX Fred Hollows^©^ and Acresoft Alcon^©^. All the IOLs’ had similar properties being aspheric, unloaded, square edge, hydrophilic, single piece with UV filter. Based on the cost of IOLs implanted patients were categorized into Group-A implanted unbranded lens (market price of PKR 1000- 3000), Group-B implanted Tecresoft model FLEX (market value of PKR 4000 - 9000PKR) and Group-C implanted Acresoft lens (market value of above PKR 10,000).

IOL selection was purely done by the patients themselves on the basis of their economic feasibility. Surgeries were allocated to various surgeons as per scheduled rota.

To reduce the factor of surgeon bias, we isolated the data of one surgeon who did maximum surgeries and have the experience of more than 20 years (the most experienced) as compare to other surgeons. This data was labelled as senior surgeon (SR) in the analysis. On the other hand, rest of the patients were operated by consultants including residents on duty in the operation theatre and this data was labelled as rest of the surgeons (RS) in the analysis.

The records are stratified for age, gender, pre and post- operative best corrected visual acuity (BCVA). Follow ups after 1^st^ day, 7^th^ day and 4-6 weeks of surgery were noted. BCVA was categorized according to “International Statistical Classification of Diseases.^13^ In this recommendation, BCVA of 6/6-6/12 is considered good, 6/18 as mild visual impairment, < 6/18 to 6/60 as moderate visual impairment and < 6/60 as severe visual impairment. Improvement, decline and stability of post-operative vision from the baseline were also expressed in n (%).

### Statistical Analysis

Data analysis was done by using Statistical Package of Social Sciences (SPSS) V 22.0. The data of BCVA was found non-parametric after checking normality through shapiro wilk test.^14^ Median and interquartile range (IQR) was calculated for BCVA (logMAR), Frequency and percentages were calculated for categorical variables like gender and Mean±S.D was calculated for age. Kruskal Wallis test^15^ was applied to check significance of post-operative visual outcomes among three categories of IOLs. P-value ≤ 0.05 was considered as significant.

## RESULTS

We were able to retrieve data of 3237 patients out of which 1589 (49%) were male. Mean age of the patients was 55.8 ± 10.9 years (minimum 17 years - maximum 95 years). All Patients underwent phacoemulsification cataract surgery followed by IOL implantation. Most of the patients, 2647 (81.8%), had IOL A. While, 254 (7.8%) had lens B and 336 (10.4%) patients had IOL C respectively. Baseline characteristics among the IOL groups, (Table-I).

**Table-I:** Baseline Characteristics of patients.

Descriptive	Group-A (N=2647)	Group-B (N=254)	Group-C (N=336)	Overall (N=3237)
Age (Mean±S.D)	55.88±11.03	56.6±9.78	55.08±11.87	55.85±10.86
Min	17	21	18	17
Max	95	85	85	95
***Gender***				
Male	1261 (47.6)	131 (51.5)	197 (58.6)	1589 (49.08)
Female	1386 (52.3)	123 (48.4)	139 (41.3)	1648 (50.91)
Baseline VA Median(IQR)	1.28(0.7)	1.04(0.5)	1.13(0.6)	1.15(0.6)

**Fig.1:**
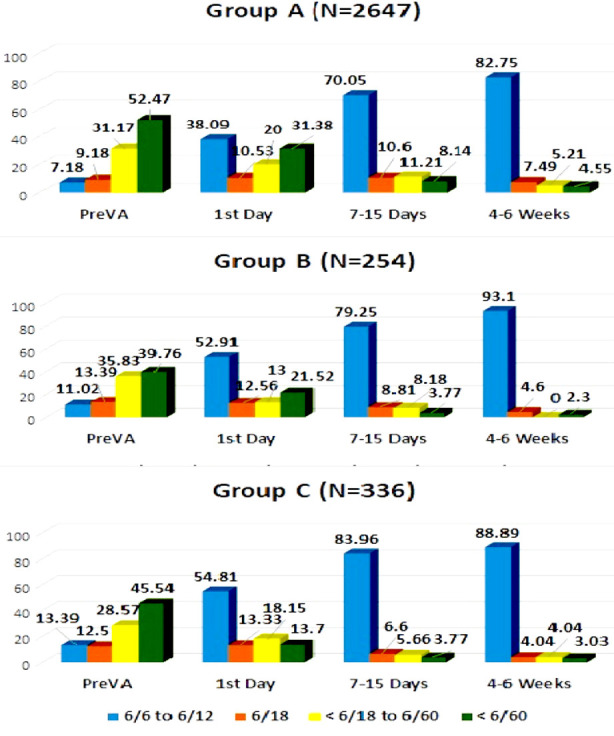
Pre and post-operative visual outcomes among three groups.

In category “A” IOL, 1394 (70.05%) achieved Best Corrected visual Acuity of 6/12 or better at 2^nd^ follow up visit improving to 873 (82.75%) on 4-6-week visit. The category “B IOL” achieved Best Corrected visual Acuity of 6/12 or better in 126 (79.3%) on 2^nd^ visit and 81 (93%) on the final visit. The category C achieved 6/12 or better in 178 (84%) at 2^nd^ visit and 88 (89%) on the final visit, Fig.1.

Comparable results were also noted in terms of visual improvements from preoperative level in 2202 (87.35%), 195 (84.4%) and 259 (88.70%) in “A”, “B” and “C” categories IOLs respectively. Similarly, visual decline was noted in 173 (6.9%), 18 (7.8%) and 14 (4.8%) in “A”, “B” and “C” categories IOL respectively. BCVA stayed stable in 5.8%, 7.8% and 19 (6.5%) in “A”, “B” and “C” categories IOL respectively (p > 0.05), (Table-II).

**Table-II:** Visual improvement and Decline.

Group-A			Group-B			Group-C			p-value

Status	Count	%	Status	Count	%	Status	Count	%	
Improve	2202	87.35	Improve	195	84.42	Improve	259	88.70	0.419
Decline	173	6.86	Decline	18	7.79	Decline	14	4.79	
Stable	146	5.79	Stable	18	7.79	Stable	19	6.51	
Total	2521	100	Total	231	100	Total	292	100	

### For Overall Surgeons (SR+RS)

A Kruskal-Wallis H test showed that there was a statistically significant difference in final follow up BCVA among three IOls, χ2(2) = 20.358, p = 0.001, with median (IQR) is 0.30 (0.2) logMAR for Group-A, 0.1 (0.3) logMAR for Group-B and 0.2 (0.1) logMAR for Group-C respectively.

### For Rest of the surgeons (RS)

A Kruskal-Wallis H test showed that there was a significant difference at final follow up BCVA among three IOLs operated by rest of the surgeons, χ2_(2)_ = 6.661, p = 0.036, with median(IQR) is 0.3(0.2) logMAR for Economical, 0.2(0.1) logMAR for Moderate and 0.2(0.3) logMAR for Expensive Lens.

### For Senior Surgeons (SR)

A Kruskal-Wallis H test showed that there was no significant difference at final follow up BCVA among three IOls operated by Senior surgeon, χ2_(2)_ = 3.216, p = 0.20, with median (IQR) is 0.2(0.2) logMAR for Group-A, 0.1(0.2) logMAR for Group-B and 0.2(0.1) logMAR for Group-C. Surgeon experience and surgical expertise is a considerable variable here, because the only calculated outcome is BCVA.

## DISCUSSION

WHO recommended at least 90% of patients have good visual outcomes (6/6 to 6/18) and not more than 5% have poor result (< 6/60) following cataract surgery in any service provision.^16^ In the present study visual outcomes of three lenses of different prices with similar qualities were compared. On average 88.2% patients in the study achieved BCVA of 6/12 or better on final follow up. When different lenses were analyzed, variable results were noted in different lenses after 4-6 weeks. The highest frequency of good BCVA (6/6 to 6/12 were achieved in patients implanted with lens B (93%), followed by lens C (89%) and lens A (82.75%) respectively.

**Chart.1:**
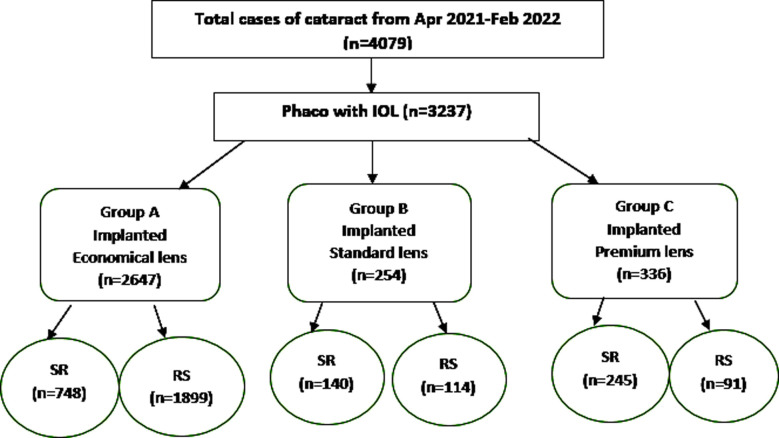
Flow Chart.

Since after the ancient ‘couching’ technique in the 5^th^ century BC, cataract surgical procedure is in constant evolution.^17^ ICCE remained the preferred method till 1970 that has been essentially replaced by ECCE. In the recent past, ECCE techniques have evolved further to the modern PHACO. Both ECCE and phaco have been followed by intra ocular lens implant IOL during last half a century. There have been innumerable studies comparing one method with the other. The technology comes with the price. Broyles et al.^18^ claimed high cost of Phacoemulsification is because of use of expensive equipment and costly consumables. One of the essential consumable in phacoemulsification cataract surgery is intraocular implants. Unfortunately, with increase branding of IOLs, current economic turmoil and high inflation is increasing cost of surgery day by day.

This problem of cost of IOL has been addressed by manufacturing low cost IOL which can bring the technology within the reach of non-affording people. There remains an ethical problem. Is the low cost IOL as effective and as safe as the branded and costly IOL? The present study was carried out to solve this problem. This study shows that branding is not very important as far as visual acuity is concerned when the surgical factors are minimized.

A study from Faisalabad reported that low cost IOLs can give as good result as high cost lenses in good surgical hands,^19^ Good visual acuity (6/6 to 6/12) was achieved in 80% and poor vision (6/60 to FC) in 8%. This study used rigid IOL and had a small sample size. A study from India compares three monofocal lenses.^20^ This study compares three monofocal lenses; Matrix Aurium, AcrySof single piece and Acrysof IQ. This study focus is more on the quality irrespective of the cost of the lens. Another Indian study,^21^ Yadev and associates compares low cost indigenous IOL (Acriol EC) with an imported aspheric IOL (AcrySof IQ) and reaches the conclusion that low cost local lens is comparable to branded high cost lens.

### Limitations

Present study has certain limitations thus warranted careful interpretation. Due to secondary source of the data, retrospective analysis we had limited control over variables. We were only able to compare BCVA between the IOLs. We were unable to do subjective analysis such as patient satisfaction survey. We were also unable to compare other objective test such as glare testing and contrast sensitivity, as they are not done routinely in clinical setups. Additionally, the data presented was collected from one center thus the results may not be generalized. This, thus warrants a prospective randomized double blind study to involve detailed assessment of all possible visual objective tests, as well as, detailed subjective assessment by using patient satisfaction survey.

## CONCLUSION

Our study shows that branding is not very important as far as visual acuity is concerned when the surgical factors are minimized. The ophthalmologist can very well be within the limits of professional ethics in advising unbranded lenses to reduce the economical burden.

### Author’s Contribution:

**MSM:** Introduction and discussion writing.

**TM:** Design of study, statistical analysis and result write up.

**SA:** Discussion writing, literature search and final review.

**UQ:** Review from clinical point of view as an ophthalmologist.

**MA:** Methodology writing.

**MFF:** Final review and assist in statistical analysis.
